# TEAS, DHEA, CoQ10, and GH for poor ovarian response undergoing IVF-ET: a systematic review and network meta-analysis

**DOI:** 10.1186/s12958-023-01119-0

**Published:** 2023-07-18

**Authors:** Fengya Zhu, Shao Yin, Bin Yang, Siyun Li, Xia Feng, Tianyu Wang, Deya Che

**Affiliations:** 1 Acupuncture and Tuina School, Chengdu University of Traditional Chinese Medicine, Chengdu, China; 2 Zigong First People’s Hospital, Zigong, China; 3 Hospital of Chengdu University of Traditional Chinese Medicine, Chengdu, China

**Keywords:** Poor ovarian response, IVF-ET, Assisted reproductive technology, NMA, Systematic review

## Abstract

**Background:**

Assisted reproductive technology (ART) has brought good news to infertile patients, but how to improve the pregnancy outcome of poor ovarian response (POR) patients is still a serious challenge and the scientific evidence of some adjuvant therapies remains controversial.

**Aim:**

Based on previous evidence, the purpose of this systematic review and network meta-analysis was to evaluate the effects of DHEA, CoQ10, GH and TEAS on pregnancy outcomes in POR patients undergoing in vitro fertilization and embryo transplantation (IVF-ET). In addition, we aimed to determine the current optimal adjuvant treatment strategies for POR.

**Methods:**

PubMed, Embase, The Cochrane Library and four databases in China (CNKI, Wanfang, VIP, SinoMed) were systematically searched up to July 30, 2022, with no restrictions on language. We included randomized controlled trials (RCTs) of adjuvant treatment strategies (DHEA, CoQ10, GH and TEAS) before IVF-ET to improve pregnancy outcomes in POR patients, while the control group received a controlled ovarian stimulation (COS) regimen only. This study was reported in accordance with the Preferred Reporting Items for Systematic Reviews and Meta-Analyses (PRISMA). The surface under the cumulative ranking curve (SUCRA) was used to provide a pooled measure of cumulative ranking for each outcome.

**Results:**

Sixteen RCTs (2323 women) with POR defined using the Bologna criteria were included in the network meta-analysis. Compared with the control group, CoQ10 (OR 2.22, 95% CI: 1.05 to 4.71) and DHEA (OR 1.92, 95% CI: 1.16 to 3.16) had obvious advantages in improving the clinical pregnancy rate. CoQ10 was the best in improving the live birth rate (OR 2.36, 95% CI: 1.07 to 5.38). DHEA increased the embryo implantation rate (OR 2.80, 95%CI: 1.41 to 5.57) and the high-quality embryo rate (OR 2.01, 95% CI: 1.07 to 3.78) and number of oocytes retrieved (WMD 1.63, 95% CI: 0.34 to 2.92) showed a greater advantage, with GH in second place. Several adjuvant treatment strategies had no significant effect on reducing the cycle canceling rate compared with the control group. TEAS was the least effective of the four adjuvant treatments in most pooled results, but the overall effect appeared to be better than that of the control group.

**Conclusion:**

Compared with COS regimen, the adjuvant use of CoQ10, DHEA and GH before IVF may have a better clinical effect on the pregnancy outcome of POR patients. TEAS needs careful consideration in improving the clinical pregnancy rate. Future large-scale RCTs with direct comparisons are needed to validate or update this conclusion.

**Systematic review registration:**

PROSPERO CRD42022304723

**Supplementary Information:**

The online version contains supplementary material available at 10.1186/s12958-023-01119-0.

## Introduction

Infertility is a common reproductive disease. Although a large number of in vitro Fertilization and Embryo Transplantation (IVF-ET) technologies have been carried out worldwide, the clinical pregnancy rate is only 30–40% [1–3]. During controlled ovarian ovarian stimulation (COS), the incidence of poor ovarian response (POR) is approximately 9–24% [4–6] and the proportion continues to increase [7, 8]. Studies have shown that the cumulative pregnancy rate of POR patients in repeated IVF-ET cycles is only 10–20% [9], which seriously affects the quality of life of patients and requires a large amount of medical resources.

POR is a pathological condition in which the ovary shows a low response to exogenous gonadotropin stimulation, mainly manifested by a decrease in the number and quality of mature oocytes obtained and a decrease in the number of transferable embryos. There are more than 40 criteria used to define POR [10], but no consensus has been reached. In 2011, the European Society of Human Reproduction and Embryology (ESHRE) proposed the Bologna standard [11]. In most subsequent studies, Bologna criteria have been widely used to define POR. For decades, modern medicine has adopted a variety of interventions to improve the outcome of IVF in POR patients, but the results are not ideal [12]. For example, increased gonadotropin use did not result in a higher pregnancy rate [13]. GnRH analogues did not significantly differ between the number of oocytes obtained and the clinical pregnancy rate [14]. Patients with POR did not benefit substantially from natural cycle IVF and the cumulative live birth rate per patient did not exceed 8% [15]. Therefore, some drug intervention before COS has become an important way to enhance the effect of exogenous gonadotrophins and improve the number and quality of oocytes. The commonly used adjuvant interventions include recombinant LH, letrozole, dehydroepiandrosterone, testosterone, growth hormone, clomiphene, estradiol, hCG, clomiphene and aspirin, among others [8].

Previous studies have directly compared the efficacy and safety of adjuvant treatment strategies for POR [7, 14, 16, 17]. However, traditional meta-analysis can only compare direct evidence and cannot determine the most effective treatment measures for patients with POR. Network meta-analysis (NMA) enables the comparison of direct and indirect evidence for multiple treatment measures in a statistical model [18, 19]. In 2020, an NMA involving 19 RCTs and 2677 POR patients assessed the impact of multiple adjuvant treatment strategies on pregnancy rates in POR patients undergoing IVF. The results showed that COS regimens with adjuvant DHEA, CoQ10 and GH showed better clinical outcomes in achieving pregnancy and required a lower dose of gonadotropin for ovulation [20]. Here, we propose another adjunctive treatment measure, transcutaneous electrical acupoint stimulation (TEAS). TEAS, which uses low-frequency pulsed current to generate electrical stimulation through electrodes attached to acupoints, is a noninvasive, painless and safe treatment technique compared with traditional acupuncture [21, 22] and is commonly used to improve IVF-ET pregnancy outcomes.

At present, RCTs continue to increase and the scientific evidence supporting these adjuvant treatments remains controversial and needs to be updated. Therefore, this study selected four adjuvant treatment strategies: DHEA, CoQ10, GH and TEAS for NMA; DHEA, CoQ10 and GH are based on previous evidence [20]. We tried to analyze direct and indirect evidence through NMA to determine the best adjuvant treatment strategy for current POR patients and provide evidence-based support.

## Methods

### Inclusion criteria

#### Study design and participants

The protocol for this manuscript has been registered in advance on the PROSPERO platform (CRD42019147503). We will report the literature in accordance with the Preferred Reporting Items for Systematic Reviews and Meta-Analyses (PRISMA) [23]. In order to obtain data directly, only published randomized controlled trials with full text available were included in this study. Subjects were POR patients undergoing IVF-ET and POR was strictly defined using the Bologna criteria. Bologna criteria: (a) advanced maternal age (≥ 40 years) or any other risk factor for POR; (b) a previous POR (≤ 3 oocytes with a conventional stimulation protocol); and (c) an abnormal ovarian reserve test (i.e. AFC < 5–7 follicles or AMH < 0.5–1.1 ng/ml). POR can be diagnosed if at least two of the three characteristics above are met. If POR occurs after two cycles of maximum ovarian stimulation regimen, POR can also be diagnosed directly.

#### Interventions

DHEA, CoQ10, GH and TEAS were used as adjuvant treatment therapies in the experimental group, all of which could be combined with the COS regimen. The drug dose, drug type, acupoint selection, frequency, waveform and cycle of each RCT are not limited. The control group only received the COS regimen. Comparisons of different doses, points or durations of the same treatment, or the use of two or more adjuvant treatments in the same group were excluded.

#### Outcomes

The primary outcome was clinical pregnancy rate; secondary outcomes included: (1) embryo implantation rate; (2) high-quality embryo rate; (3) cycle canceling rate; (4) live birth rate; and (5) number of oocytes retrieved. Eligible RCTs included at least the primary outcome clinical pregnancy rate, otherwise they were excluded. Clinical pregnancy was defined as the presence of a gestational sac or fetal heartbeat using ultrasound [24].

### Search strategy

PubMed, Embase, The Cochrane Library and four databases in China (CNKI, Wanfang, VIP and SinoMed) were systematically searched up to July 30, 2022, with no language restrictions. Both MeSH terms and text terms were used in the literature search to identify potential RCTs and the search strategy was adjusted according to different databases (Supplementary Table S1). In addition, we performed a manual search of relevant references to identify additional eligible studies.

### Study selection and data extraction

Two reviewers independently searched and screened the literature according to the search strategy. Before the data were retrieved, the disputed RCT was discussed by the reproductive experts to determine whether it would eventually be incorporated in the analysis. Two reviewers then independently extracted the basic characteristics of each RCT according to a standardized form, including first author, publication year, language, sample size, age, diagnostic criteria, intervention details and outcomes. All of the above differences were discussed and a consensus was reached by two people. The differences were resolved by the third reviewer.

### Quality assessment of risk of bias

Two reviewers used the Cochrane handbook to evaluate each risk of bias (RoB). Assessments included random sequence generation, allocation concealment, blinding of participants and staff, blinding of outcome assessments, incomplete outcome data, selective reporting and other biases. Each area was rated as low risk, high risk, or unclear. Any discrepancies were determined by a third reviewer.

### Data synthesis and statistical analysis

NMA analysis was performed using Stata15.1, where the odds ratio (OR) and 95% confidence interval (95% CI) were used for dichotomous data and weighted mean difference (WMD) and 95% CI were used for continuous data. The network graph is used to show the results of direct comparisons of different interventions between studies. The size of nodes in the network graph is the sample size of the intervention and the thickness of lines between nodes represents the number of RCTs directly compared between the two interventions. Then, global and local inconsistency were evaluated (node-splitting method). If P > 0.05, this indicated that there was no significant difference in the estimated effect size between direct and indirect comparisons and the consistency model was used. We used the surface under the cumulative ranking curve (SUCRA) to provide aggregated measures of cumulative ranking for each outcomes, with the results presented as percentages. For direct data, pairwise meta-analysis was performed using a random-effects model and the OR value was converted to number of treatments required (NNT) to account for clinical pregnancy rate, embryo implantation rate, high-quality embryo rate, cycle canceling rate and live birth rate. Finally, we used adjusted comparison funnel plots to assess the impact of small studies.

## Results

### Study selection and characteristics

**Fig. 1:**
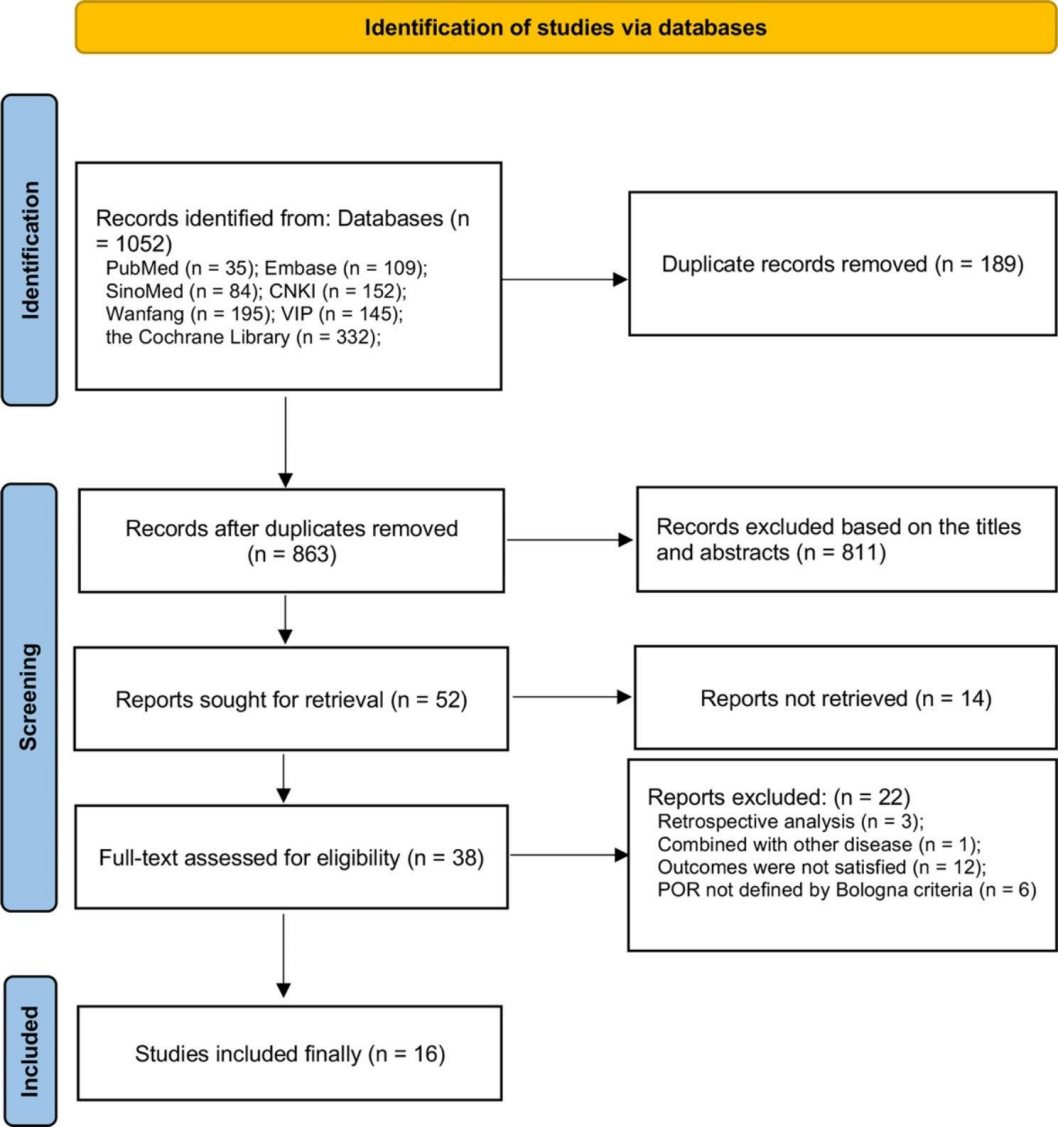
The screening flow chart

A total of 1052 publications were retrieved from 7 databases, with 189 duplicates excluded as well as 811 papers according to the title and abstract. After further reading the full text, 16 RCTs [25–40] were finally included in this study (see Fig. 1 for the detailed flow chart). Supplementary Table S2 shows the exclusion list and reasons. All 16 RCTs were single-center studies, including 11 in China, 3 in Egypt and the remaining two in Iran and South Korea. Sample sizes for individual RCTs ranged from 38 to 821 and all studies used Bologna diagnostic criteria to define POR. The 16 RCTs included 2323 participants with POR undergoing IVF who were randomly assigned to receive four different adjuvant therapies, including DHEA, GH, CoQ10 and TEAS. The control group was the COS regimen and the specific characteristics of the study are shown in Table 1.

**Table 1:** Characteristics of included studies

Included studies	Country	Sample size (I/C)	Duration of infertility [y, mean (SD)] (I/C)	BMI [kg/m2] (I/C)	Age [y, mean (SD)] (I/C)	Diagnostic criteria	Intervention (dose/duration)	Control	Outcomes
Hu R, 2018	China	41/37	5(4.60)/4.67(3.84)	21.67(2.81)/22.45(3.12)	37.33 (3.07)/36.67 (3.86)	Bologna	DHEA (25 mg, tid/3 m)	FSH 300 ~ 450 IU/d, on day 2 ~ 3 of the menstrual cycle; HCG 6000 ~ 10,000 IU	①②③④
Li XL, 2018	China	32/40	4.18(2.95)/4.66(2.93)	22.70(2.86)/21.94(2.79)	33.13 (4.70)/32.85 (5.31)	Bologna	GH (4 IU, qd/ day 2–3 of the menstrual cycle t to the day of HCG)	one week after ovulation, triptorelin, 0.1 mg/d and changed to 0.5 mg/d after 1–2 weeks; HCG 10,000 IU	①②③⑥⑤
Liao CR, 2017	China	56/47	7.25(3.85)/8.29(3.42)	——	37.90 (3.08)/ 38.05 (2.35)	Bologna	DHEA (25 mg, tid/3 m)	Gonal-F, 150 ~ 225 U/d, on day 3 of the menstrual cycle; Cetrorelix, 0.25 mg/d; HCG 10,000 IU	①④⑥
Song H, 2015	China	56/56	5.85(2.12)/6.28(1.83)	——	37.14 (5.21)/36.86 (5.72)	Bologna	DHEA (25 mg, tid/3 m)	HCG 6000 ~ 10000U	①②③④⑥
Tang Y, 2013	China	21/17	9.6(1.1)/7.6(0.8)	——	34.5 (0.7)/32.8 (1.2)	Bologna	GH (4 IU, qd/ day 2–3 of the menstrual cycle t to the day of HCG)	triptorelin, 0.05 mg/d, 1 ~ 2 day after ovulation; Gonal-F, 300IU/d, 2 ~ 3 day after ovulation; HCG 5000 ~ 10,000 IU	①
Wu XY, 2018	China	48/48	3.5(1.50)/3.7(1.10)	24.1(2.60)/23.7(1.80)	36.4 (3.70)/37.3 (2.90)	Bologna	GH (4.5 IU, qd/ day 2–3 of the menstrual cycle t to the day of HCG)	Gn, 225 ~ 375 IU/d; GnRH-A, 0.25 mg/d, on day 2 ~ 3 of the menstrual cycle; HCG, 250 mg	①②⑥
Xu Y, 2018	China	76/93	3(1.54)/2.67(0.76)	21.85(2.51)/22.24(3.07)	32.50 (3.30)/31.92 (3.68)	Bologna	CoQ10(200 mg, tid/ 60 d)	Gonal-F and HMG, both 225 IU/d, on day 2 of the menstrual cycle; Cetrorelix 250 μg/d; Ovidrele 250 μg	①⑤⑥
Safdarian L, 2019	Iran	34/26	3.71(1.63)/3.16(1.62)	26.43(3.24)/26.63(3.07)	33.80 (4.66)/33.91 (4.49)	Bologna	GH (2.5 mg, qd/ day 8 of the menstrual cycle t to the day of HCG)	Gonal-F, 300 ~ 450 IU/d, on day 3 of the menstrual cycle; Cetrotide, 0.25 mg/d; HCG 10,000 IU	①⑥⑤
Gong Y, 2020	China	52/53	4.57(3.14)/4.39(3.22)	22.09(1.73)/22.62(2.73)	38.41 (2.91)/38.20 (2.79)	Bologna	GH (4 IU, qd/ day 2 of the menstrual cycle t to the day of HCG)	Gonal-F/on day 2 of the menstrual cycle; Ganirelix; HCG	①②③④⑥
Mi H, 2014	China	32/32	4.31 (0.60)/ 4.75(0.58)	23.11 (0.73)/ 24.31(0.53)	38 (0.92)/ 37.8 (0.55)	Bologna	TEAS (2 Hz, 20–25 mA, 30 min, qd/ 3 m)	Estradiol valerate tablets/2 mg po qd, 21 days, on the fifth day of menstruation; Dydrogesterone Tablets/10 mg po bid, 5 days, 3 periods	①②③④⑥
Lian F, 2017	China	46/46	4.03(1.62)/4.28(1.57)	——	40.18 (2.82)/ 40.06 (2.62)	Bologna	TEAS (2 Hz, 20–25 mA, 30 min, qd/ 1 m)	HMG, 150 ~ 300 IU/d, on day 2 ~ 4 of menstruation; Cetrorelix Acetate, 0.125 ~ 0.25 mg/d; HCG 5000 ~ 10,000 IU	①③⑥
Bassiouny YA, 2016	Egypt	68/73	6.44(3.62)/6.75(3.75)	23.71(5.06)/22.67(3.71)	35.79 (5.56)/35.53 (5.98)	Bologna	GH (7.5 IU, qd/ day 6 of hMG stimulation until the day of hCG triggering)	HMG, 300–450 IU/d, from the second day of the cycle; Cetrotide, 0.25 mg/d; HCG 10,000 IU	①②⑤
Wang Z, 2021	China	410/411	3.33(3.72)/3.17(3.72)	23.83(3.16)/24.01(3.07)	39.00 (4.64)/39.53 (4.39)	Bologna	DHEA (25 mg, tid/4–12 w)	Triptorelin, 0.05 ~ 0.1 mg/on day 2 ~ 3 of menstruation; Menotropins, 150 ~ 225 IU/d; HCG 4000 ~ 10,000 IU	①⑤
Kotb MMM, 2016	Egypt	70/70	7.9(2.5)/7.6(2.6)	25.6(3.4)/25.1(3.4)	40.05 (3.1)/39.7 (0.5)	Bologna	DHEA (25 mg, tid/3 m)	HMG, 300 ~ 450 IU/d, on the second day of menstruation; Cetrotide 0.25 mg/d; HCG 10,000 IU	①④
Choe SA, 2018	South Korea	62/65	3.85(2.69)/4.49(3.57)	21.2(2.5)/21.1(2.4)	39.8 (3.6)/39.4 (4.1)	Bologna	GH (20 mg/ three times, mid-luteal, late luteal, and menstrual cycle day 2)	Gonal-F, 225 ~ 375 IU/from menstrual day 3; Cetrotide, 0.25 mg/d; HCG	①③⑥
Mohammad EH, 2021	Egypt	78/78	6.62(2.13)/6.35(2.01)	24.39(1.52)/25.06(3.47)	34.27 (2.41)/34.74 (1.98)	Bologna	GH (4 IU,qd/ from the second day of the cycle and stopped one day before ovum pickup)	Cetrorelix, 0.25 mg/d, started on day 6 of COH; HCG 10,000 IU	①②

I, intervention group; C, control group; w, week; m, month; qd, once a day; tid, hree times a day; DHEA, dehydroepiondrosterone; GH, Growth hormone. FSH, follicle stimulating hormone; HCG, human chorionic gonadotropin; COH, controlled ovarian hyperstimulation; HMG, human menopausal gonadotropin

①: Clinical pregnancy rate; ②: Embryo implantation rate; ③: High-quality embryo rate; ④: Cycle canceling rate; ⑤: Live birth rate; ⑥: Number of oocytes retrieved

### Risk of bias

The risk of bias of 16 RCTs was assessed in Supplementary Figure S1. In terms of random sequence generation, 13 RCTs (81.2%) had a low risk of bias. Six RCTs (37.5%) had a low risk of bias in terms of allocation concealment. However, only 4 RCTs (25%) had a low risk of bias on the two assessments associated with blinding. All studies were low risk in terms of data integrity and selective reporting.

### Network meta-analysis results

#### Primary outcomes: clinical pregnancy rate

**Fig. 2:**
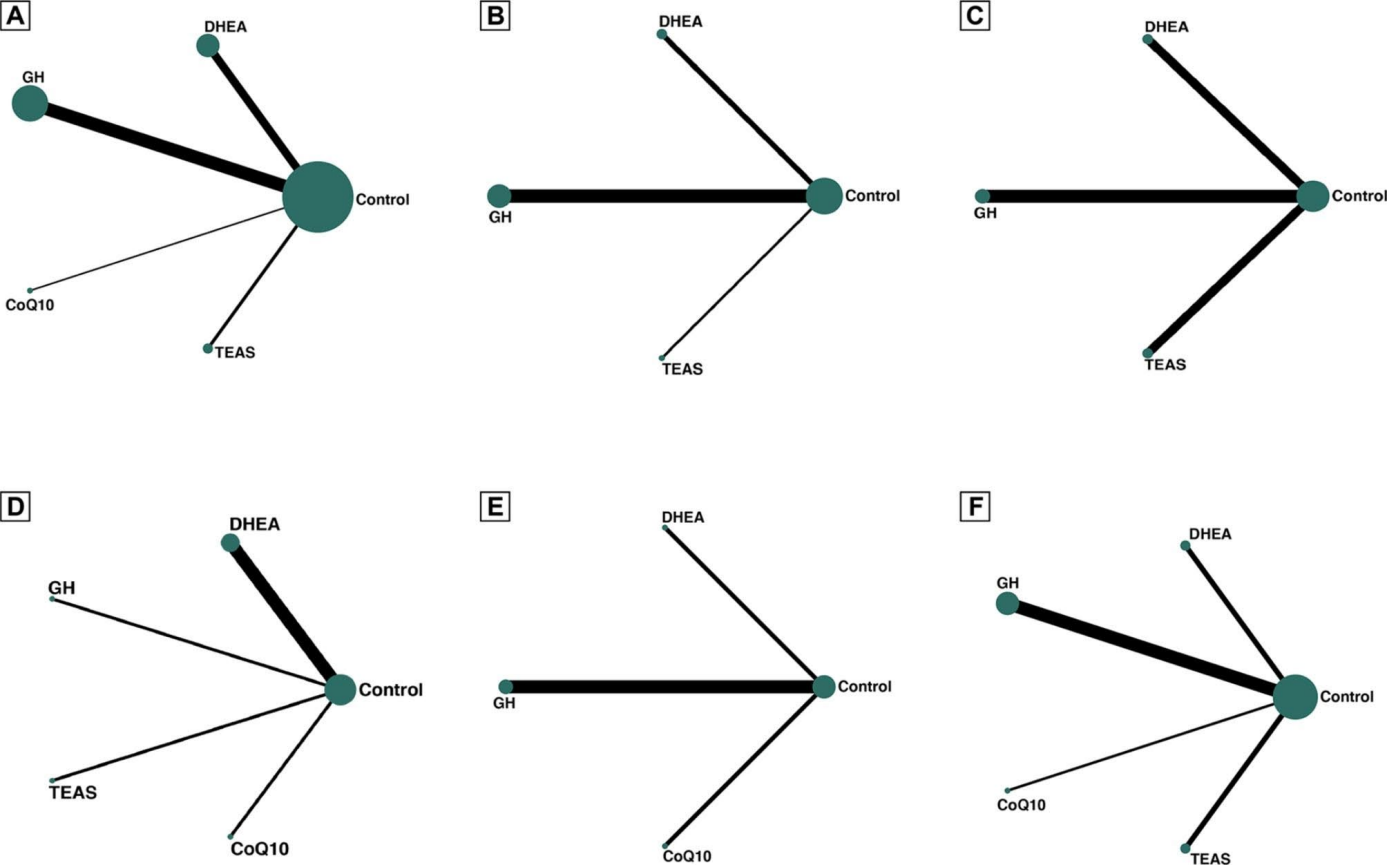
The network plot (**A** Clinical pregnancy rates; **B** Embryo implantation rate; **C** High-quality embryo rate; **D** Cycle canceling rate; **E** Live birth rate; **F** Number of oocytes retrieved)

**Fig. 3:**
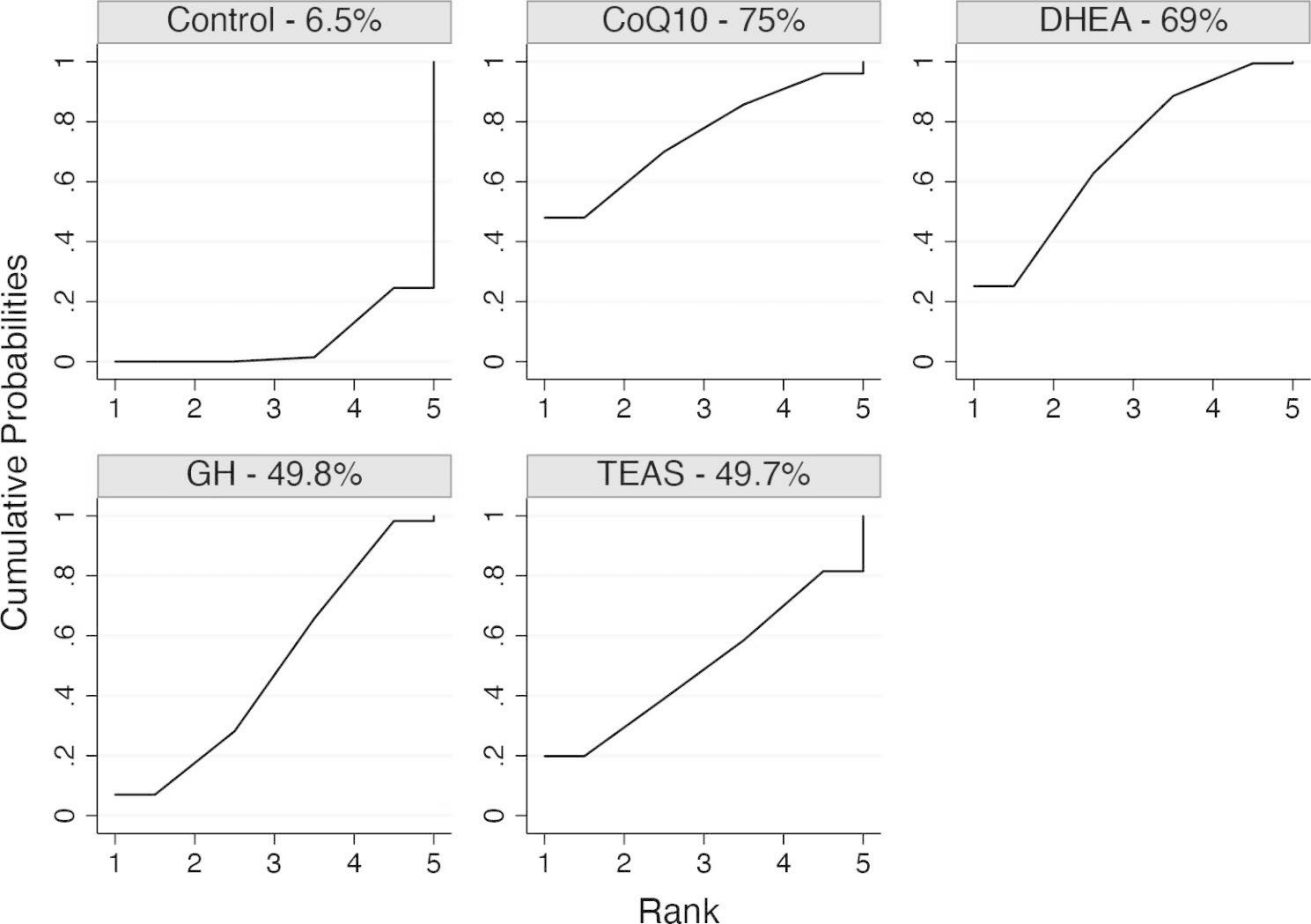
SUCRA analysis: MeanRank figure (Clinical pregnancy rate)

A total of 16 RCTs (2323 participations) reported clinical pregnancy rates. The network plot is shown in Fig. 2A. Due to the lack of inconsistent resources, we used a consistent model. Compared with the control group, CoQ10 (OR 2.22, 95%CI: 1.05 to 4.71) and DHEA (OR 1.92, 95%CI: 1.16 to 3.16) showed obvious advantages in improving the clinical pregnancy rate. The results of network meta-analysis are shown in Table 2. The SUCRA values of CoQ10, DHEA, GH, TEAS and the control were 75%, 69%, 49.8%, 49.7% and 6.5%, respectively (Fig. 3and Supplementary Table S3). The pairwise meta-analysis results conducted with direct data were basically consistent with the above results. Detailed results and NNTs are provided in Supplementary Table S4. The results of the adjusted comparison funnel plot showed that the small-sample study had no effect on the clinical pregnancy rate (Supplementary Figure S2).

**Table 2:** Odds ratio (OR) with 95% confidence interval on Clinical pregnancy rate

**CoQ10**				
1.16 (0.41,3.26)	**DHEA**			
1.42 (0.52,3.89)	1.23 (0.64,2.37)	**GH**		
1.43 (0.37,5.45)	1.23 (0.41,3.71)	1.00 (0.34,2.95)	**TEAS**	
**2.22 (1.05,4.71)**	**1.92 (1.16,3.16)**	1.56 (0.99,2.58)	1.56 (0.58,4.18)	**Control**

#### Secondary outcomes: embryo implantation rate

Eight RCTs (773 participations) reported on the embryo implantation rate; the network plot is shown in Fig. 2B. Due to the lack of inconsistent resources, we used a consistent model. The results of network meta-analysis are shown in Supplementary Table S5. Compared with the control group, DHEA (OR 2.80, 95%CI: 1.41 to 5.57) and GH (OR 1.60, 95%CI: 1.09 to 2.36) can better improve the embryo implantation rate.The SUCRA values of DHEA, GH, TEAS and control were 87.1%, 57.1%, 53.8% and 8%, respectively (Supplementary Figure S3 and Supplementary Table S6). The paired meta-analysis also showed the same results (Supplementary Table S4).

#### Secondary outcomes: high-quality embryo rate

A total of 7 RCTs involving 650 participations were included in the network meta-analysis of high-quality embryo rate (see Fig. 2C for the network plot). Due to a lack of inconsistent resources, we used a consistent model. A total of three auxiliary measures were compared with the control group. The results of network meta-analysis showed that DHEA (OR 2.01, 95%CI: 1.07 to 3.78) was associated with a higher rate of high-quality embryos (Supplementary Table S7). The SUCRA values of DHEA, GH, TEAS and the control were 88.1%, 60.1%, 53.1% and 16%, respectively (Supplementary Figure S4 and Supplementary Table S8). The results of pairwise meta-analysis based on direct data were basically consistent with the results of mesh meta-analysis (Supplementary Table S4).

#### Secondary outcomes: cycle canceling rate

Seven RCTs (771 participations) explored the effect of adjuvant therapy on cycle canceling rate. See Fig. 2D for the network plot. Due to a lack of inconsistent resources, we used a consistent model. Compared with the control group, the four auxiliary measures of DHEA, GH, CoQ10 and TEAS had no obvious advantage in reducing the cycle canceling rate (Supplementary Table S9). The SUCRA values of GH, CoQ10, DHEA, TEAS and control were 81.0%, 69.9%, 49.5%, 30.9% and 18.8%, respectively (Supplementary Figure S5 and Supplementary Table S10). Finally, the paired meta-analysis also showed the same results (Supplementary Table S4).

#### Secondary outcomes: live birth rate

A total of 5 RCTs (1263 participations) were involved in the live birth rate, as shown in Fig. 2E for the network plot. Due to the lack of inconsistent resources, we used a consistent model. CoQ10 (OR 2.36, 95%CI: 1.07 to 5.38) can effectively improve the live birth rate (Supplementary Table S11). The SUCRA values of CoQ10, GH, control and DHEA were 89.9%, 63.6%, 23.8% and 22.7%, respectively (Supplementary Figure S6 and Supplementary Table S12). The paired meta-analysis results were basically consistent with the above results (Supplementary Table S4).

#### Secondary outcomes: number of oocytes retrieved

Regarding the effect of the four adjunctive interventions on the number of oocytes retrieved, a total of 10 RCTs involved 949 participants (see Fig. 2F for the network plot). Due to the lack of resources for discordant, we therefore used a concordant model. Compared with the control group, the results of network meta-analysis showed that DHEA (WMD 1.63, 95%CI: 0.34 to 2.92), GH (WMD 1.50, 95%CI: 0.61 to 2.39), CoQ10 (WMD 1.34, 95%CI 0.64 to 1.99), TEAS (WMD 1.04, 95%CI: 0.24 to 3.02) four auxiliary measures could increase the number of oocytes retrieved (Supplementary Table S13). DHEA, GH, CoQ10, TEAS and the Control of SUCRA values were 72.2%, 67.6%, 59%, 47.5% and 3.7% (Supplementary Figure S7 and Supplementary Table S14). Finally, we conducted a pairwise meta-analysis based on direct data; the results were the same as those of mesh analysis, as shown in Supplementary Table S4.

## Discussion

### Discussion of the main results

To improve IVF pregnancy outcomes in POR patients and determine the best adjuvant treatment strategy, we sought to update the clinical evidence. In this study, we conducted indirect and direct comparisons of CoQ10, DHEA, GH, TEAS and conventional COS regimens, evaluating several clinical outcomes of most concern in the field of reproductive medicine. We found that: [1] CoQ10 was significantly better than DHEA, GH, TEAS and control in improving clinical pregnancy rates and live birth rates; [2] DHEA showed greater advantages in improving the embryo implantation rate, high-quality embryo rate and the number of oocytes retrieved; [3] these adjuvant treatment measures have no significant effect on reducing the cycle canceling rate; and [4] in most pooled results, TEAS had the worst efficacy of the four adjuvant treatments, but the overall effect seemed to be better than that of the control group.

Limited by the Bologna criteria, only one RCT with CoQ10 as adjuvant therapy was included in this study [31]. Analysis of the data obtained revealed that CoQ10 treatment had the highest live birth rate (89.9%), followed by clinical pregnancy rate (75%). CoQ10, as an antioxidant, has been used to improve infertility outcomes, which is associated with increased clinical pregnancy rate (CPR), although the quality of previous evidence remains low [41]. Consistent with our results, a systematic review showed that CoQ10 supplementation significantly increased the clinical pregnancy rate in women with POR and PCOS [42]. In addition, our findings show that CoQ10 was the only statistically significant intervention of the four adjuvant therapies to improve the live birth rate. Despite the limited clinical evidence, CoQ10 has promising applications in adjuvant therapy strategies for POR patients. Of course, these results need to be confirmed by further well-designed prospective RCTs with a large number of participants.

The current meta-analysis included five RCTs with DHEA for POR. Adjuvant treatment with DHEA has a significant impact on various pregnancy outcomes. Our results showed that DHEA produced better clinical outcomes in terms of improving the embryo implantation rate, the high-quality embryo rate and the number of oocytes retrieved. This study suggests that POR patients undergoing IVF improved the conditions of early pregnancy after taking DHEA, which seems to imply an indirect increase in the clinical pregnancy rate. In some direct comparative evidence, DHEA supplementation had a positive effect on women with reduced ovarian reserve (DOR) or POR undergoing IVF/ICSI [43–46], which can improve the ovarian environment for follicle maturation [17]. Compared with placebo or untreated women, the use of DHEA improved the live-birth rate and the ongoing pregnancy rate increased by 3–14% [47]; its mechanism was through the effect on granular cell and ovarian matrix expression of androgen receptor and it also increased the quantity of follicular cavity and AMH level, thereby increasing ovarian reserve [48]. In contrast, several studies have shown that DHEA use is not associated with higher clinical pregnancy rates [49–52], although some scholars suggest that large-scale confirmatory studies are needed to prove the efficacy of DHEA before recommending its routine use [53, 54]. While we support the view of Gleicher and Barad [55]; However, in the case of insufficient available evidence, whether it is possible to supplement the use of DHEA to enhance the effect of exogenous gonadotropins in suitable POR patients to improve ovarian reserve and potential pregnancy outcomes. Further evidence is necessary.

GH has been widely used to treat infertility, especially for patients with POR and the rationale is based on animal and human data. GH may increase the production of insulin-like growth factor 1 (IGF-1) in the ovary. IGF-1 is believed to play an important role in regulating ovarian function [56, 57], stimulating follicle development [58], improving oocyte quality [59] and promoting estrogen production and oocyte maturation [57]. The results of multiple meta-analyses confirmed the beneficial effect of GH on clinical outcomes, including increasing the number of oocytes retrieved, the number of MII oocytes and the number of transferable embryos, thus improving the clinical pregnancy rate and live birth rate. At the same time, the cycle canceling rate and gonadotropin dose of POR patients were decreased [7, 14, 60, 61]. Eight RCTs were evaluated, with GH second only to DHEA in terms of improving the number of oocytes retrieved and the high-quality embryo rate, but there was no significant benefit in improving the clinical pregnancy rate and live birth rate. The Cochrane systematic review noted that because the doses and regimens of GH in the trials were variable, the effects on pregnancy outcomes were uncertain and results needed to be interpreted with caution [62, 63]. However, prospective, large-scale clinical studies may also have different diagnostic criteria and COS protocols, which could increase the risk of bias and imprecision. Therefore, further studies are needed to fully determine the role of GH as an adjunctive treatment for IVF.

TEAS is a non-invasive and painless hybrid therapy that combines transcutaneous electrical nerve stimulation with traditional Chinese medicine acupuncture [21]. TEAS has recently formed a consensus group in reproductive medicine [64]. Multiple RCTs showed that TEAS significantly increased the embryo implantation rate, clinical pregnancy rate and live birth rate [65, 66], improved the basic endocrine level and endometrial receptivity of patients and increased the number of embryos and high-quality embryos [67, 68]. However, in the current meta-analysis, TEAS did not show a better effect in improving pregnancy outcomes in POR patients and did not differ significantly compared with controls. However, the SUCRA value showed that the overall efficacy of combined TEAS in the conventional regimen was better than that of the conventional regimen. Inconsistencies with clinical findings may involve various variable parameters of TEAS, such as frequency, acupoint selection, treatment cycle and even operator level, which may be important factors affecting the clinical effect. Therefore, the potential value of TEAS still needs to be further validated.

### Strengths and limitations of the study

Our systematic review and network meta-analysis strictly used the Bologna criteria to define POR, which minimized the risk of heterogeneity and bias. Second, we used network meta-analysis to rank multiple treatment measures in a statistical model [18, 19] and to cover several important clinical outcomes including: (1) embryo implantation rate; (2) high-quality embryo rate; (3) cycle canceling rate; (4) live birth rate; and (5) number of oocytes retrieved. These results can provide a reference for the selection of clinical adjunct regimens for POR.

There are some limitations to our study. There has been no consensus on the definition of POR; after the ESHRE organization proposed the Bologna criteria in 2011, some experts pointed out that the detailed definition of some risk factors had not been solved and the population was very heterogeneous [69]. In 2016, the POSEIDON team proposed a new stratification method based on the number and quality of oocytes, called the POSEIDON criterion [70], but carrying out large RCTs with this criterion can be difficult. First of all, it must be said that our strict use of Bologna criteria to define patients with POR limited the inclusion of some RCTs; only 16 RCTs were included in the network meta-analysis, indirect evidence and the overall risk of bias in the included studies is not optimistic, which may have affected our judgment of the overall quality. Second, there was a gap in the number of RCTs of the four adjuvant therapies, including GH (8 RCTs), DHEA (5 RCTs), TEAS (2 RCTs) and CoQ10 (1 RCT). Due to the limited number of studies, we could not conduct a detailed subgroup analysis according to factors such as adjuvant therapy and exogenous gonadotropin use, initiation time and treatment cycle to reduce heterogeneity. Third, we did not perform an analysis of adverse events due to insufficient primary data; no study provided long-term follow-up data, such as infant growth and development, due to the high time and economic costs involved. Finally, most of the included studies were from Asia or the Middle East, whether population differences had an impact on the outcomes remains to be improved in subsequent studies. Based on current clinical evidence, the clinical efficacy of TEAS remains controversial, but this non-invasive nerve stimulation technique developed from acupoints may provide some new ideas for the treatment of infertility. It may be helpful to reassess the effectiveness of TEAS by summarizing a protocol for prescribing acupoints or standardizing the formation of surface stimulation points.

## Conclusion

According to the current evidence, CoQ10, DHEA and GH adjuvant therapy before IVF may have a positive effect on pregnancy outcome in POR patients compared with the conventional COS regimen. TEAS was the worst at improving clinical pregnancy rates, even though it was a noninvasive ex vivo intervention. Infertility patients form a large population worldwide and future large-scale RCTs with direct comparisons are needed to validate or update this conclusion.

## Electronic supplementary material

Below is the link to the electronic supplementary material.

**Supplementary Material** 
Supplementary Table S1: Search strategy
Supplementary Table S2: Exclusion list
Supplementary Table S3: SUCRA analysis: MeanRank table (Clinical pregnancy rate)
Supplementary Table S4: Pairwise meta-analysis results for direct comparisons of outcomes
Supplementary Table S5: Odds ratio (OR) with 95% confidence interval on Embryo implantation rate
Supplementary Table S6: SUCRA analysis: MeanRank table (Embryo implantation rate)
Supplementary Table S7: Odds ratio (OR) with 95% confidence interval on High-quality embryo rate)
Supplementary Table S8: SUCRA analysis: MeanRank table (High-quality embryo rate)
Supplementary Table S9: Odds ratio (OR) with 95% confidence interval on Cycle canceling rate
Supplementary Table S10: SUCRA analysis: MeanRank table (Cycle canceling rate)
Supplementary Table S11: Odds ratio (OR) with 95% confidence interval on Live birth rate
Supplementary Table S12: SUCRA analysis: MeanRank table (Live birth rate)
Supplementary Table S14: SUCRA analysis: MeanRank table (Number of oocytes retrieved)
Supplementary Figure S1: Risk of bias assessment results
Supplementary Figure S3: SUCRA analysis: MeanRank figure (Embryo implantation rate)
Supplementary Figure S4: SUCRA analysis: MeanRank figure (High-quality embryo rate)
Supplementary Figure S5: SUCRA analysis: MeanRank figure (Cycle canceling rate)
Supplementary Figure S6: SUCRA analysis: MeanRank figure (Live birth rate)
Supplementary Figure S7: SUCRA analysis: MeanRank figure (Number of oocytes retrieved)

## Data Availability

Data can be obtained from authors on reasonable request.
