# Supplementary material for: TEAS, DHEA, CoQ10, and GH for poor ovarian response undergoing IVF-ET: a systematic review and network meta-analysis
Source: Reprod Biol Endocrinol. 2023 Jul 18;21:64. doi: 10.1186/s12958-023-01119-0 (PMC10355041; doi:10.1186/s12958-023-01119-0)
Supplement: Supplementary file 1 — Supplementary Material Supplementary Table S1: Search strategy Supplementary Table S2: Exclusion list Supplementary Table S3: SUCRA analysis: MeanRank table (Clinical pregnancy rate) Supplementary Table S4: Pairwise meta-analysis results for direct comparisons of outcomes Supplementary Table S5: Odds ratio (OR) with 95% confidence interval on Embryo implantation rate Supplementary Table S6: SUCRA analysis: MeanRank table (Embryo implantation rate) Supplementary Table S7: Odds ratio (OR) with 95% confidence interval on High-quality embryo rate) Supplementary Table S8: SUCRA analysis: MeanRank table (High-quality embryo rate) Supplementary Table S9: Odds ratio (OR) with 95% confidence interval on Cycle canceling rate Supplementary Table S10: SUCRA analysis: MeanRank table (Cycle canceling rate) Supplementary Table S11: Odds ratio (OR) with 95% confidence interval on Live birth rate Supplementary Table S12: SUCRA analysis: MeanRank table (Live birth rate) Supplementary Table S14: SUCRA analysis: MeanRank table (Number of oocytes retrieved) Supplementary Figure S1: Risk of bias assessment results Supplementary Figure S3: SUCRA analysis: MeanRank figure (Embryo implantation rate) Supplementary Figure S4: SUCRA analysis: MeanRank figure (High-quality embryo rate) Supplementary Figure S5: SUCRA analysis: MeanRank figure (Cycle canceling rate) Supplementary Figure S6: SUCRA analysis: MeanRank figure (Live birth rate) Supplementary Figure S7: SUCRA analysis: MeanRank figure (Number of oocytes retrieved) [file 12958_2023_1119_MOESM1_ESM.docx]

# Supplementary Table S1: Search strategy

1. **PubMed Database**

#1 Dehydroepiandrosterone[MeSH Terms] OR DHEA[All Fields] OR Growth hormone[MeSH Terms]) OR GH[All Fields] OR coenzyme Q10[All Fields] OR CoQ10[All Fields] OR transcutaneous electrical acupoint stimulation[All Fields] OR transcutaneous acupoint electrical stimulation[All Fields] OR TEAS[All Fields] OR TAES[All Fields]

#2 Fertilization in Vitro[MeSH Terms] OR Sperm Injections, Intracytoplasmic[All Fields] OR in vitro fertilization[All Fields] OR in-vitro fertilization[All Fields] OR IVF[All Fields] OR ICSI[All Fields] OR intra-cytoplasmic sperm injection[All Fields] OR intracytoplasmic sperm injection[All Fields] OR Embryo Transfer[All Fields] OR ET[All Fields]

#3 poor ovarian response[MeSH Terms] OR poor ovarian responder[All Fields] OR poor responders[All Fields] OR poor responder[All Fields] OR diminished ovarian reserve[All Fields] OR poor prognostic patients[All Fields] OR low prognosis[All Fields] OR poor prognosis[All Fields]

#4 randomized controlled trial[Publication Type] OR controlled clinical trial[Publication Type] OR clinical trials[Publication Type] OR randomly[Publication Type] OR Randomized[Publication Type]

#5 #1 AND #2 AND #3 AND #4

#### Embase Database

#1 ('dehydroepiandrosterone':ti,ab,kw OR 'dhea':ti,ab,kw OR 'growth hormone':ti,ab,kw OR 'gh':ti,ab,kw OR 'coenzyme q10':ti,ab,kw OR coq10:ti,ab,kw OR 'transcutaneous electrical acupoint stimulation':ti,ab,kw OR 'transcutaneous acupoint electrical stimulation':ti,ab,kw OR 'TEAS':ti,ab,kw OR 'TAES':ti,ab,kw)

#2 ('fertilization in vitro':ti,ab,kw OR 'sperm injections, intracytoplasmic':ti,ab,kw OR 'in vitro fertilization':ti,ab,kw OR 'in-vitro fertilization':ti,ab,kw OR ivf:ti,ab,kw OR icsi:ti,ab,kw OR 'intra- cytoplasmic sperm injection':ti,ab,kw OR 'intracytoplasmic sperm injection':ti,ab,kw OR 'embryo transfer':ti,ab,kw OR et:ti,ab,kw)

#3 ('poor ovarian response':ti,ab,kw OR 'poor ovarian responder':ti,ab,kw OR 'poor responders':ti,ab,kw OR 'poor responder':ti,ab,kw OR 'diminished ovarian reserve':ti,ab,kw OR 'poor prognostic patients':ti,ab,kw OR 'low prognosis':ti,ab,kw OR 'poor prognosis':ti,ab,kw)

#4 ('randomized controlled trial':ti,ab,kw OR 'controlled clinical trial':ti,ab,kw OR 'clinical trials':ti,ab,kw OR 'randomly':ti,ab,kw OR 'randomized':ti,ab,kw)

#5 #1 and #2 and #3 and #4

1. **the Cochrane Library Database** #1 Dehydroepiandrosterone: MeSH #2 (DHEA):ti,ab,kw

#3 growth hormone: MeSH #4 (GH):ti,ab,kw

#5 (Coenzyme Q10):ti,ab,kw

#6 CoQ10: MeSH

#7 (transcutaneous electrical acupoint stimulation):ti,ab,kw #8 (transcutaneous acupoint electrical stimulation):ti,ab,kw #9 (TEAS):ti,ab,kw

#10 (TAES):ti,ab,kw

#11 #1 OR #2 OR #3 OR #4 OR #5 OR #6 OR #7 OR #8 OR #9 OR #10

#12 Fertilization in Vitro: MeSH

#13 (Sperm Injections, Intracytoplasmic):ti,ab,kw #14 (in vitro fertilization):ti,ab,kw

#15 (in-vitro fertilization):ti,ab,kw

#16 (IVF):ti,ab,kw

#17 (ICSI):ti,ab,kw

#18 (intra-cytoplasmic sperm injection):ti,ab,kw #19 (intracytoplasmic sperm injection):ti,ab,kw #20 (Embryo Transfer):ti,ab,kw

#21 (ET):ti,ab,kw

#22 #12 OR #13 OR #14 OR #15 OR #16 OR #17 OR #18 OR #19 OR #20 OR #21

#23 (poor ovarian response):ti,ab,kw #24 (poor ovarian responder):ti,ab,kw #25 (poor responders):ti,ab,kw

#26 (diminished ovarian reserve):ti,ab,kw #27 (poor prognostic patients):ti,ab,kw #28 (low prognosis):ti,ab,kw

#29 (poor prognosis):ti,ab,kw

#30 #23 OR #24 OR #25 OR #26 OR #27 OR #28 OR #29

#31 (randomized controlled trial):pt #32 (controlled clinical trial):pt

#33 (clinical trials):pt

#34 (randomly):pt

#35 (Randomized):pt

#36 #31 OR #32 OR #33 OR #34 OR #35

#38 #11 AND #22 AND #30 AND #36

#### SinoMed Database

("CoQ10"[common field: intelligent] AND "growth hormone "[common field: intelligent] OR "DHEA"[common field: intelligent] OR" percutaneous acupoint electrical stimulation "[common field: intelligent] OR "TEAS"[common field: intelligent]) AND (" Low ovarian response "[common field: intelligent]) and (" Low ovarian response "[common field: intelligent] OR "Low ovarian function "[Common fields: Intelligence] OR" low ovarian reserve "[common field: intelligent] OR" low ovarian response "[common field: intelligent] OR" low ovarian reserve "[common field: intelligent] OR" POR"[common field: intelligent]) AND (" randomized control "[common field: intelligent] OR "Clinical research" [common fields: intelligent] OR "effect" [common fields: intelligent] OR "observe" [common fields: intelligent] OR "comparison" [common fields: intelligent] OR "research" [common fields: intelligent] OR "influence" [common fields: intelligent])

#### CNKI Database

SU = (' CoQ10 '+' growth hormone '+' DHEA '+' transcutaneous point electric stimulation '+' TEAS ') AND SU = (' low ovarian response '+' poor ovarian function '+' low ovarian reserve '+' low ovarian response '+' poor ovarian reserve function '+' POR ') AND SU= (' randomized controlled trial '+' clinical study '+' efficacy '+' observation '+' comparison '+' research '+' impact ')

#### VIP Database

U = (CoQ10 + growth hormone + DHEA + transcutaneous point electric stimulation + TEAS) AND U = (low ovarian response + poor ovarian function + low ovarian reserve + low ovarian response + poor ovarian reserve function + POR) AND U= (randomized controlled trial+ clinical research + efficacy + observation + comparison + research + impact)

#### Wanfang Database

Subject :(" CoQ10" or "growth hormone" or "DHEA" or "transcutaneous acupoint electrical stimulation" or "TEAS") and subject :(" low ovarian response "or" low ovarian function "or" low ovarian reserve "or" low ovarian response "or "Low ovarian reserve "or "POR") and subject :(" Randomized controlled "or" clinical study "or "Efficacy "or" observation "or "comparison "or" study "or "Impact ")

**Supplementary Table S2: Exclusion list**

#### POR not defined by Bologna criteria (n = 6)

1. Suikkari A, MacLachlan V, Koistinen R, Seppälä M, Healy D. Double-blind placebo-controlled study: human biosynthetic growth hormone for assisted reproductive technology. Fertil Steril. 1996 Apr;65(4):800-5.
2. Kucuk T, Kozinoglu H, Kaba A. Growth hormone co-treatment within a GnRH agonist long protocol in patients with poor ovarian response: a prospective, randomized, clinical trial. J Assist Reprod Genet. 2008 Apr;25(4):123-7.
3. Wiser A, Gonen O, Ghetler Y, Shavit T, Berkovitz A, Shulman A. Addition of dehydroepiandrosterone (DHEA) for poor-responder patients before and during IVF treatment improves the pregnancy rate: a randomized prospective study. Hum Reprod. 2010 Oct;25(10):2496-500.
4. Yovich JL, Stanger JD. Growth hormone supplementation improves implantation and pregnancy productivity rates for poor-prognosis patients undertaking IVF. Reprod Biomed Online. 2010 Jul;21(1):37-49.
5. Norman RJ, Alvino H, Hull LM, Mol BW, Hart RJ, Kelly TL, Rombauts L; LIGHT investigators. Human growth hormone for poor responders: a randomized placebo-controlled trial provides no evidence for improved live birth rate. Reprod Biomed Online. 2019 Jun;38(6):908-915.
6. Eftekhar M, Aflatoonian A, Mohammadian F, Eftekhar T. Adjuvant growth hormone therapy in antagonist protocol in poor responders undergoing assisted reproductive technology. Arch Gynecol Obstet. 2013 May;287(5):1017-21.

#### Outcomes were not satisfied (n = 12)

1. Yeung TW, Chai J, Li RH, Lee VC, Ho PC, Ng EH. A randomized, controlled, pilot trial on the effect of dehydroepiandrosterone on ovarian response markers, ovarian response, and in vitro fertilization outcomes in poor responders. Fertil Steril. 2014 Jul;102(1):108-115.
2. Dakhly DMR, Bassiouny YA, Bayoumi YA, Hassan MA, Gouda HM, Hassan AA. The addition of growth hormone adjuvant therapy to the long down regulation protocol in poor responders undergoing in vitro fertilization: Randomized control trial. Eur J Obstet Gynecol Reprod Biol. 2018 Sep;228:161- 165.
3. Bayoumi YA, Dakhly DM, Bassiouny YA, Hashish NM. Addition of growth hormone to the microflare stimulation protocol among women with poor ovarian response. Int J Gynaecol Obstet. 2015 Dec;131(3):305-8.
4. Howles CM, Loumaye E, Germond M, et al.Does growth hormone-releasing factor assist follicular development in poor responder patients undergoing ovarian stimulation for in-vitro fertilization? [J].Human Reproduction,1999,14(8):1939-1943.
5. Bergh C, Carlström K, Selleskog U, Hillensjö T. Effect of growth hormone on follicular fluid androgen levels in patients treated with gonadotropins before in vitro fertilization. Eur J Endocrinol. 1996 Feb;134(2):190-6.
6. Dor J, Seidman DS, Amudai E, Bider D, Levran D, Mashiach S. Adjuvant growth hormone therapy in poor responders to in-vitro fertilization: a prospective randomized placebo-controlled double-blind study. Hum Reprod. 1995 Jan;10(1):40-3.
7. Hughes SM, Huang ZH, Morris ID, Matson PL, Buck P, Lieberman BA. A double-blind cross-over controlled study to evaluate the effect of human biosynthetic growth hormone on ovarian stimulation in previous poor responders to in-vitro fertilization. Hum Reprod. 1994 Jan;9(1):13-8.
8. Huang ZH, Baxter RC, Hughes SM, Matson PL, Lieberman BA, Morris ID. Supplementary growth hormone treatment of women with poor ovarian response to exogenous gonadotrophins: changes in serum and follicular fluid insulin-like growth factor-1 (IGF-1) and IGF binding protein-3 (IGFBP-3). Hum Reprod. 1993 Jun;8(6):850-7.
9. Owen EJ, West C, Mason BA, Jacobs HS. Co-treatment with growth hormone of sub-optimal responders in IVF-ET. Hum Reprod. 1991 Apr;6(4):524-8.
10. Artini PG, Simi G, Ruggiero M, Pinelli S, Di Berardino OM, Papini F, Papini S, Monteleone P, Cela

V. DHEA supplementation improves follicular microenviroment in poor responder patients. Gynecol Endocrinol. 2012 Sep;28(9):669-73.

1. Zhou Te. Effect of acupuncture on the number of eggs retrieved and the rate of optimal embryos in patients with low ovarian response IVF microstimulation regimen [M]: Chengdu University of Traditional Chinese Medicine, 2019.
2. Jiao J, Jia T, Feng X, Sun W. Clinical observation of percutaneous electrical acupoint stimulation in 90 infertile patients with poor ovarian response [J]. China healthy birth and heredity, 2017, 25 (8) : 114- 116 + 113. DOI: 10.13404 / j.carol carroll nki CJBHH. 2017.08.047.

#### Retrospective analysis (n = 3)

1. Fusi FM, Ferrario M, Bosisio C, Arnoldi M, Zanga L. DHEA supplementation positively affects spontaneous pregnancies in women with diminished ovarian function. Gynecol Endocrinol. 2013 Oct;29(10):940-3.
2. Lee YX, Shen MS, Tzeng CR. Low Dose Growth Hormone Adjuvant Treatment With Ultra-Long Ovarian Stimulation Protocol in Poor Responders Showed Non-inferior Pregnancy Outcome Compared With Normal Responders. Front Endocrinol (Lausanne). 2019 Dec 20;10:892.
3. Gao RF, Tu ZR, Wang LY, Duan RY. Effect of dehydroepiandrosterone (DHEA) pretreatment on the clinical outcome of IVF/intracytoplasmic sperm injection-embryo transfer in patients with low ovarian response [J]. International journal of reproductive health/family planning,2019,38(05):370-373.

#### Combined with other disease (n = 1)

1.Owen EJ, Shoham Z, Mason BA, Ostergaard H, Jacobs HS. Cotreatment with growth hormone, after pituitary suppression, for ovarian stimulation in in vitro fertilization: a randomized, double-blind, placebo-control trial. Fertil Steril. 1991 Dec;56(6):1104-10.

**Supplementary Table S3: SUCRA analysis: MeanRank table (Clinical pregnancy rate).**

+ +

| Treatm~t | SUCRA | PrBest | MeanRank |

| + + + |

| \| | Control \| 6.5 \| | 0.0 \| | 4.7 | \| |
| --- | --- | --- | --- | --- |
| \| | DHEA \| 69.0 \| | 25.2 \| | 2.2 | \| |
| \| | GH \| 49.8 \| | 7.0 \| | 3.0 | \| |
| \| | CoQ10 \| 75.0 \| | 48.0 \| | 2.0 | \| |
| \| | TEAS \| 49.7 \| | 19.8 \| | 3.0 | \| |

+ +

**Supplementary Table S4: Pairwise meta-analysis results for direct comparisons of outcomes.**

| **Outcomes** | **Effect size (95% CI)** | **No. of**  **RCTs** | **I^2^ (p-value)** | **NNT** |
| --- | --- | --- | --- | --- |
| **Clinical pregnancy rate** | | | | |
| DHEA versus Control | OR 1.90 (1.21, 2.97) | 5 | 8.8% (p = 0.356) | 8.6 |
| GH versus Control | OR 1.60 (0.98, 2.61) ^*^ | 8 | 36.6% (p = 0.137) | 13.9 |
| CoQ10 versus Control | OR 2.22 (1.08, 4.58) | 1 | - | 3.9 |
| TEAS versus Control | OR 1.55 (0.63, 3.82) ^*^ | 2 | 0.0% (p = 0.640) | 6.5 |
| **Embryo implantation rate** | | | | |
| DHEA versus Control | OR 2.81 (1.45, 5.45) | 2 | 0.0% (p = 0.333) | 7.1 |
| GH versus Control | OR 1.61 (1.08, 2.38) | 5 | 11.3% (p = 0.341) | 10.2 |
| TEAS versus Control | OR 1.80 (0.39, 8.27) ^*^ | 1 | - | 11.4 |
| **High-quality embryo rate** | | | | |
| DHEA versus Control | OR 1.96 (1.01, 6.06) | 2 | 79.0% (p = 0.029) | 8.6 |
| GH versus Control | OR 1.47 (0.96, 2.25) ^*^ | 3 | 26.4% (p = 0.257) | 11.1 |
| TEAS versus Control | OR 1.13 (0.65, 1.97) ^*^ | 2 | 0.0% (p = 0.954) | 44.2 |
| **Cycle canceling rate** | | | | |
| DHEA versus Control | OR 0.71 (0.39, 1.30) ^*^ | 4 | 0.0% (p = 0.882) | 26.5 |
| CoQ10 versus Control | OR 0.46 (0.14, 1.53) ^*^ | 1 | - | 18.1 |
| GH versus Control | OR 0.34 (0.09, 1.38) ^*^ | 1 | - | 10.7 |
| TEAS versus Control | OR 1.05 (0.31, 3.57) ^*^ | 1 | - | 100 |
| **Live birth rate** | | | | |
| DHEA versus Control | OR 0.97 (0.60, 1.57) ^*^ | 1 | - | 50 |
| GH versus Control | OR 1.59 (0.58,4.37) ^*^ | 3 | 17.5% (p = 0.298) | 29.2 |
| CoQ10 versus Control | OR 2.30 (1.08, 4.89) | 1 | - | 7.5 |
| **Number of oocytes retrieved** | | | | |
| DHEA versus Control | WMD 1.63 (0.79, 2.47) | 2 | 80.6% (p = 0.023) | - |
| GH versus Control | WMD 1.51 (0.54, 2.48) | 5 | 82.6% (p = 0.000) | - |
| CoQ10 versus Control | WMD 1.34 (0.65, 2.03) | 1 | - | - |
| TEAS versus Control | WMD 1.12 (0.95, 1.29) | 2 | 0.0% (p = 0.595) | - |

^*^ *The result was not achieving statistical significance (P >0.05).*

**Supplementary Table S5: Odds ratio (OR) with 95% confidence interval on Embryo implantation rate.**

**DHEA**

### 1.56 (0.29,8.44) **TEAS**

1.75 (0.79,3.84) 1.12 (0.23,5.51) **GH**

### 2.80 (1.41,5.57) 1.80 (0.38,8.44) 1.60 (1.09,2.36) **Control**

**Supplementary Table S6: SUCRA analysis: MeanRank table (Embryo implantation rate).**

+ +

| Treatm~t | SUCRA | PrBest | MeanRank |

| + + |

| \| Control | \| 8.0 \| | 0.0 \| | 3.8 | \| |
| --- | --- | --- | --- | --- |
| \| DHEA | \| 87.1\| | 66.2 \| | 1.4 | \| |
| \| GH | \| 51.1\| | 4.6 \| | 2.5 | \| |
| \| TEAS | \| 53.8\| | 29.2 \| | 2.4 | \| |

+ +

**Supplementary Table S7: Odds ratio (OR) with 95% confidence interval on High-quality embryo rate).**

## DHEA

### 1.39 (0.61,3.16) **GH**

1.78 (0.67,4.72) 1.28 (0.51,3.18) **TEAS**

### 2.01 (1.07,3.78) 1.44 (0.85,2.45) 1.13 (0.54,2.38) **Control**

**Supplementary Table S8: SUCRA analysis: MeanRank table (High-quality embryo rate).**

+ +

| Treatm~t | SUCRA | PrBest | MeanRank |

| + + + |

| \| Control | \|16.0 \| | 0.1 \| | 3.5 | \| |
| --- | --- | --- | --- | --- |
| \| DHEA | \|88.1 \| | 72.5 \| | 1.4 | \| |
| \| GH | \|60.8 \| | 18.5 \| | 2.2 | \| |
| \| TEAS | \| 35.1 \| | 8.9 \| | 2.9 | \| |

+ +

**Supplementary Table S9: Odds ratio (OR) with 95% confidence interval on Cycle canceling rate.**

| **GH** |  |  | |
| --- | --- | --- | --- |
| 0.75 (0.12,4.68) | **CoQ10** |  |  |
| 0.49 (0.11,2.21) | 0.65 (0.17,2.51) | **DHEA** |  |
| 0.36 (0.06,2.30) | 0.49 (0.09,2.69) | 0.74 (0.19,2.91) | **TEAS** |
| 0.34 (0.09,1.38) | 0.46 (0.14,1.53) | 0.71 (0.39,1.30) | 0.95 (0.28,3.22) **Control** |

**Supplementary Table S10: SUCRA analysis: MeanRank table (Cycle canceling rate).**

+ +

| Treatm~t | SUCRA | PrBest | MeanRank |

| + + + |

| \|Control | \|18.8 \| | 0.1 \| | 4.2 | \| |
| --- | --- | --- | --- | --- |
| \|DHEA | \|49.5 \| | 5.0 \| | 3.0 | \| |
| \|GH | \|81.0 \| | 56.2 \| | 1.8 | \| |
| \|TEAS | \|30.9 \| | 5.9 \| | 3.8 | \| |
| \|CoQ10 | \|69.9 \| | 32.8 \| | 2.2 | \| |
| + |  |  |  | + |

**Supplementary Table S11: Odds ratio (OR) with 95% confidence interval on Live birth rate.**

## CoQ10

### 1.49 (0.46,4.78) **GH**

2.30 (1.08,4.89) 1.55 (0.63,3.76) **Control**

### 2.36 (1.07,5.38) 1.59 (0.58,4.37) 1.03 (0.64,1.66) **DHEA**

**Supplementary Table S12: SUCRA analysis: MeanRank table (Live birth rate).**

+ +

| Treatm~t | SUCRA | PrBest | MeanRank |

| + + + |

| \| Control | \|23.8 \| | 0.2 \| | 3.3 | \| |
| --- | --- | --- | --- | --- |
| \| DHEA | \|22.7 \| | 1.1 \| | 3.3 | \| |
| \|GH | \|63.6 \| | 25.4 \| | 2.1 | \| |
| \|CoQ10 | \|89.9 \| | 73.3 \| | 1.3 | \| |
| + |  |  |  | + |

**Supplementary TableS 13: Odds ratio (OR) with 95% confidence interval on Number of oocytes retrieved**

| **DHEA** |  |  |  | |
| --- | --- | --- | --- | --- |
| 0.13 (-1.43,1.69) | **GH** |  |  |  |
| 0.29 (-1.98,2.56) | 0.16 (-1.91,2.23) | **CoQ10** |  |  |
| 0.59 (-1.22,2.41) | 0.46 (-1.10,2.02) | 0.30 (-1.97,2.57) | **TEAS** |  |
| 1.63 (0.34,2.92) | 1.50 (0.61,2.39) | 1.34 (0.64,1.99) | 1.04 (0.24,3.02) | **Control** |

**Supplementary Table S14: SUCRA analysis: MeanRank table (Number of oocytes retrieved).**

+ +

| Treatm~t | SUCRA | PrBest | MeanRank |

| + + + |

| \| Control | \| 3.7 \| | 0.0 \| | 4.9 | \| |
| --- | --- | --- | --- | --- |
| \| DHEA | \|72.3 \| | 37.0 \| | 2.1 | \| |
| \| GH | \|67.6 \| | 24.2 \| | 2.3 | \| |
| \| CoQ10 | \|59.0 \| | 28.3 \| | 2.6 | \| |
| \| TEAS | \|47.5 \| | 10.5 \| | 3.1 | \| |
| + |  |  |  | + |

**Supplementary Figure S1: Risk of bias assessment results** (A: Assessment of each risk of bias item presented as percentages across all included RCTs; B: Assessment of each risk of bias item for each included RCT).


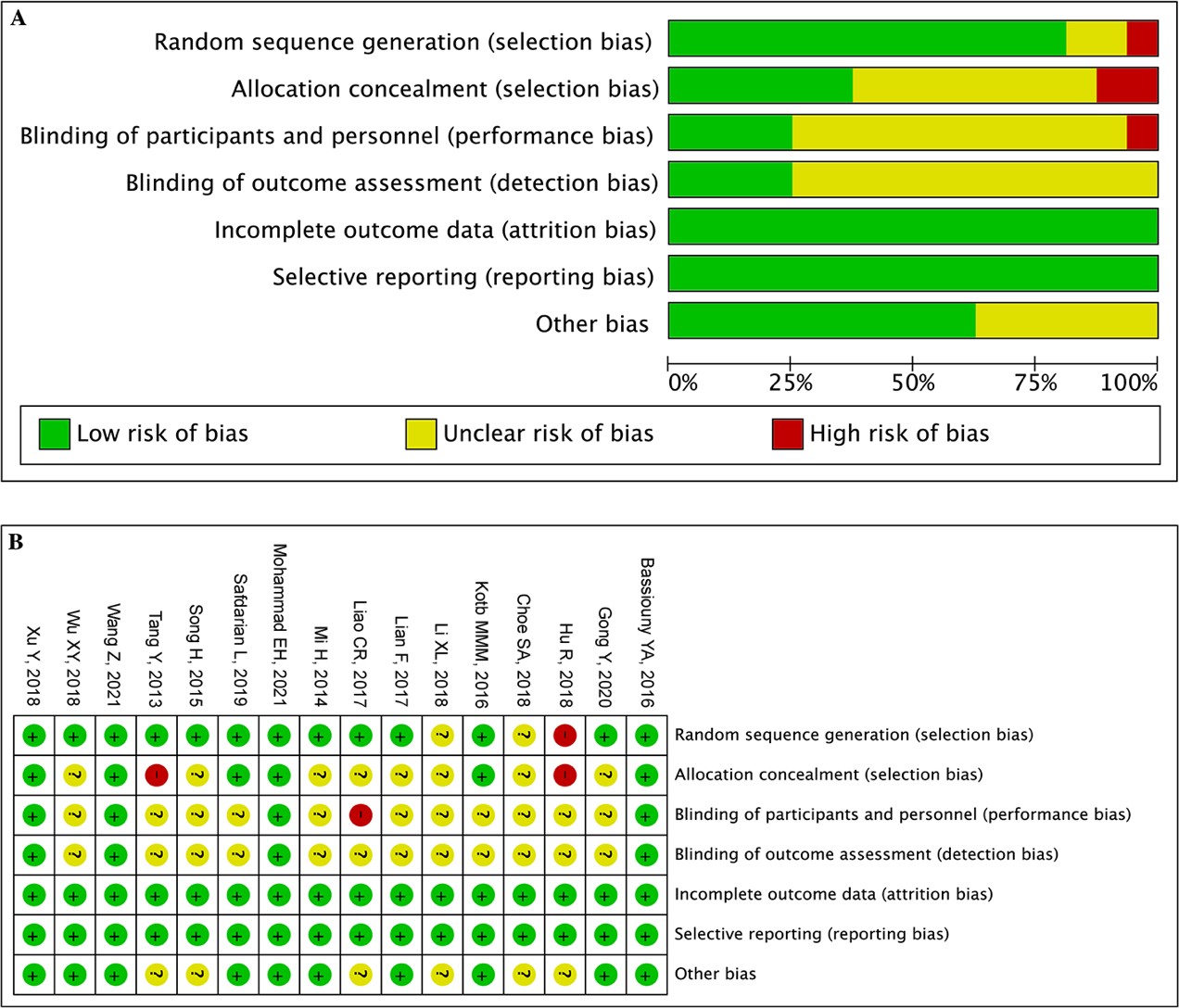


# Supplementary Figure S2: Comparison-adjusted funnel plot for the clinical pregnancy rate.


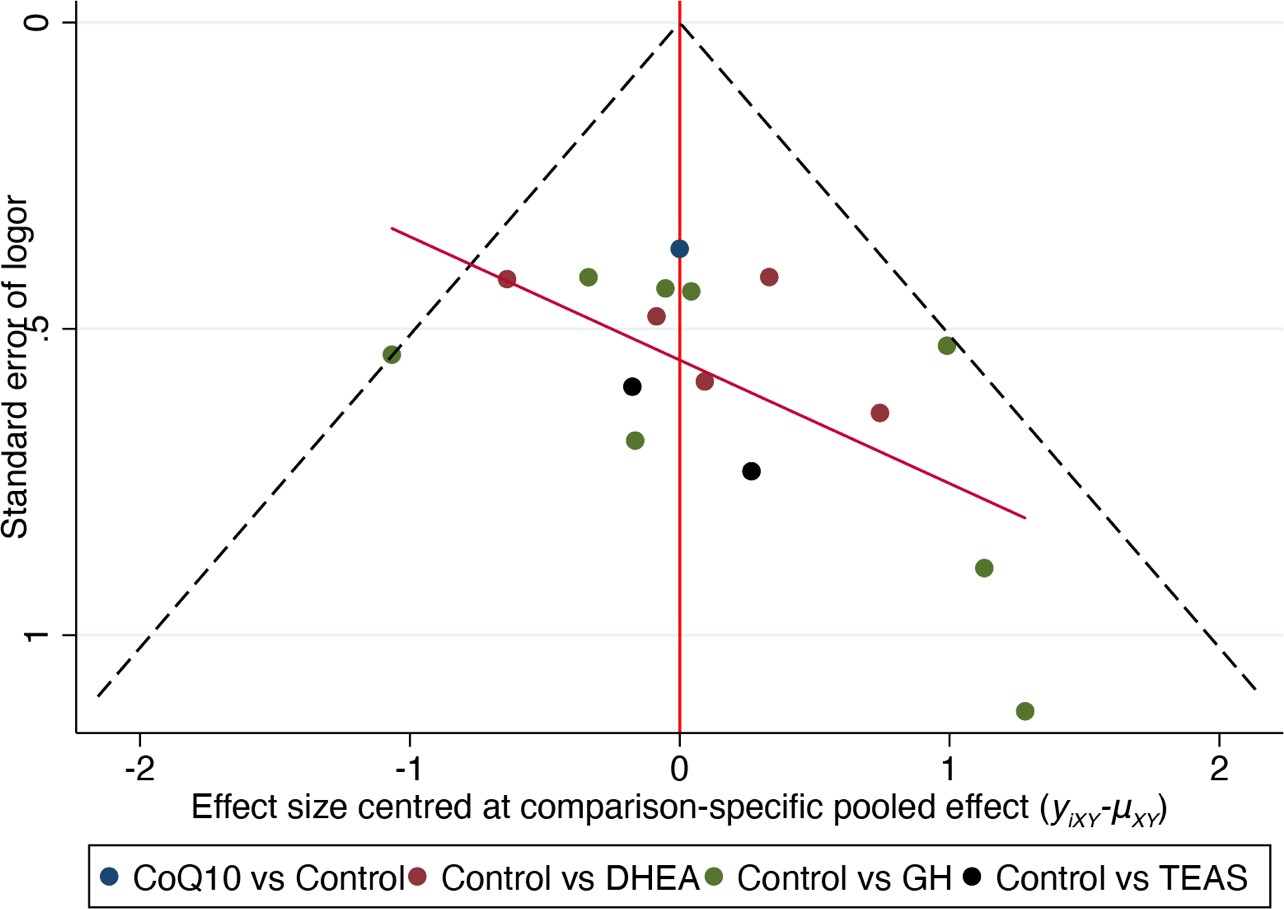


**Supplementary Figure S3: SUCRA analysis: MeanRank figure (Embryo implantation rate).**


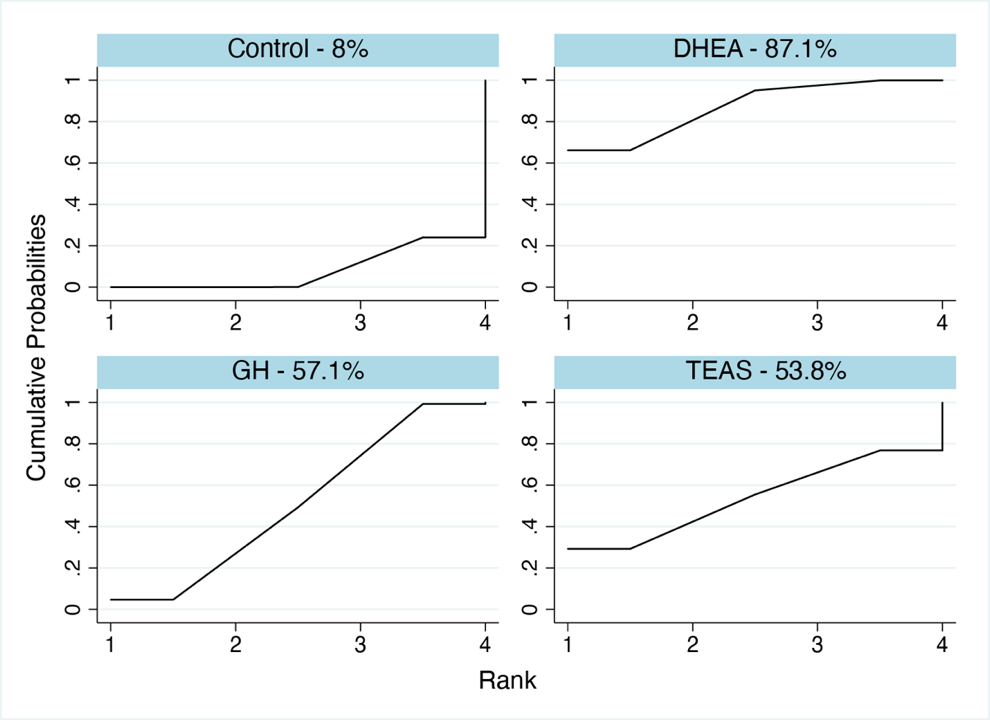


**Supplementary Figure S4: SUCRA analysis: MeanRank figure (High-quality embryo rate).**


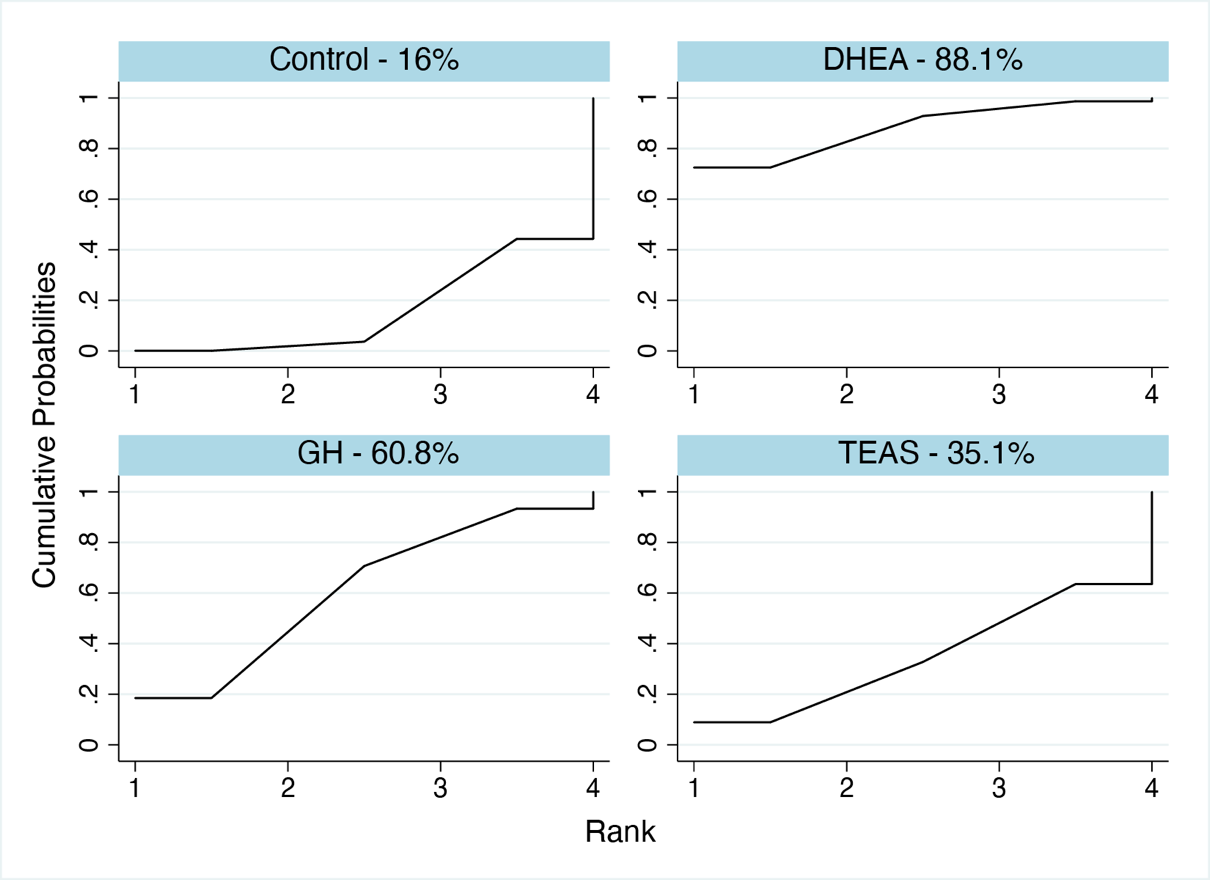


# Supplementary Figure S5: SUCRA analysis: MeanRank figure (Cycle canceling rate).

**
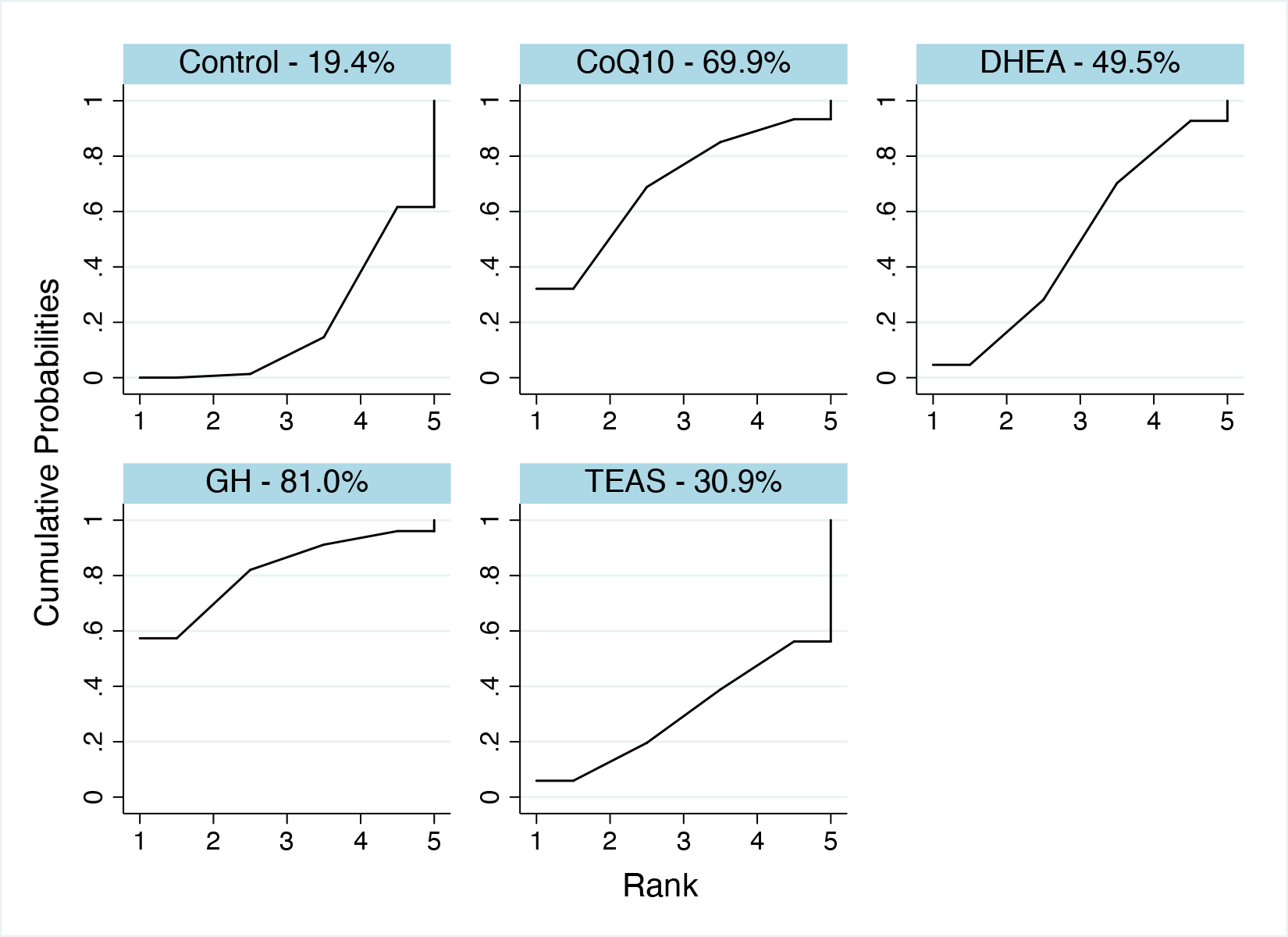
**

**Supplementary Figure S6: SUCRA analysis: MeanRank figure (Live birth rate).**


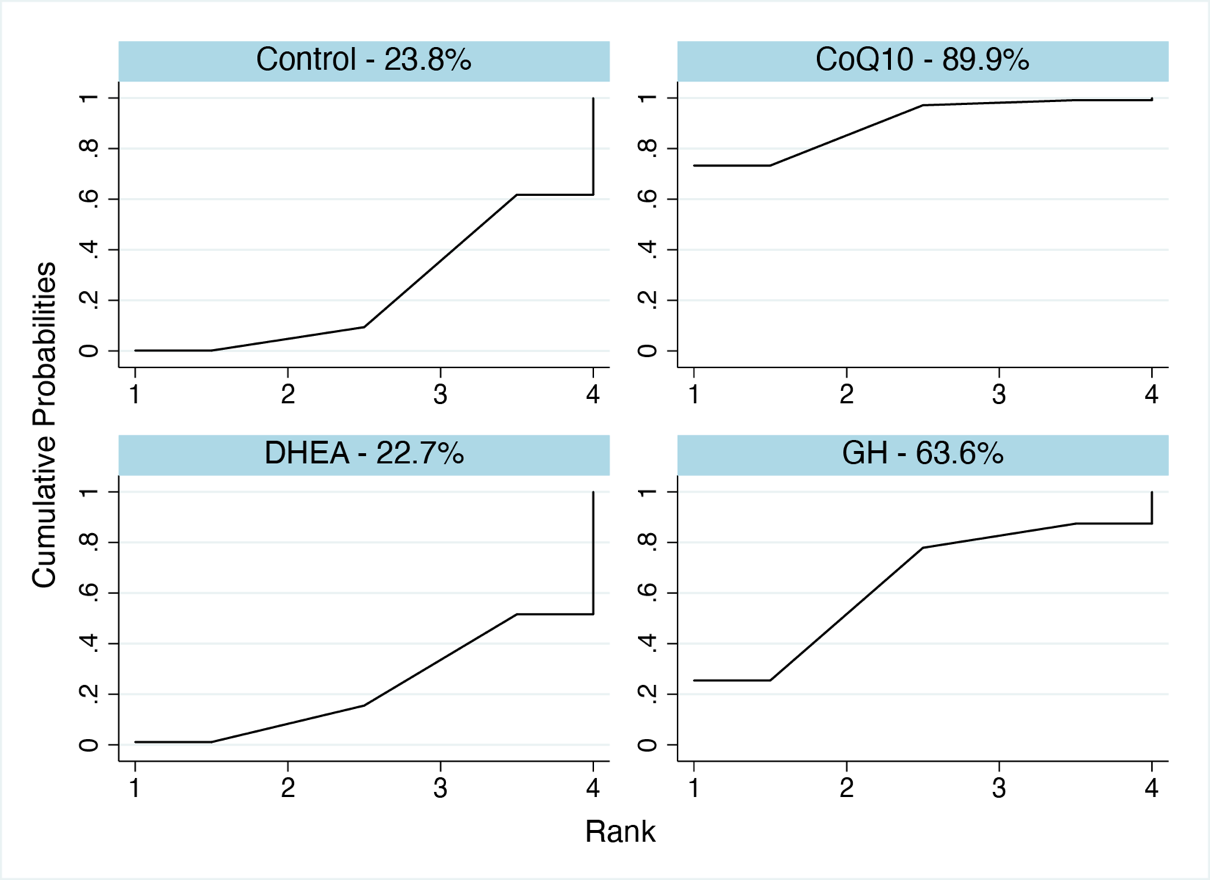


# Supplementary Figure S7: SUCRA analysis: MeanRank figure (Number of oocytes retrieved).

**
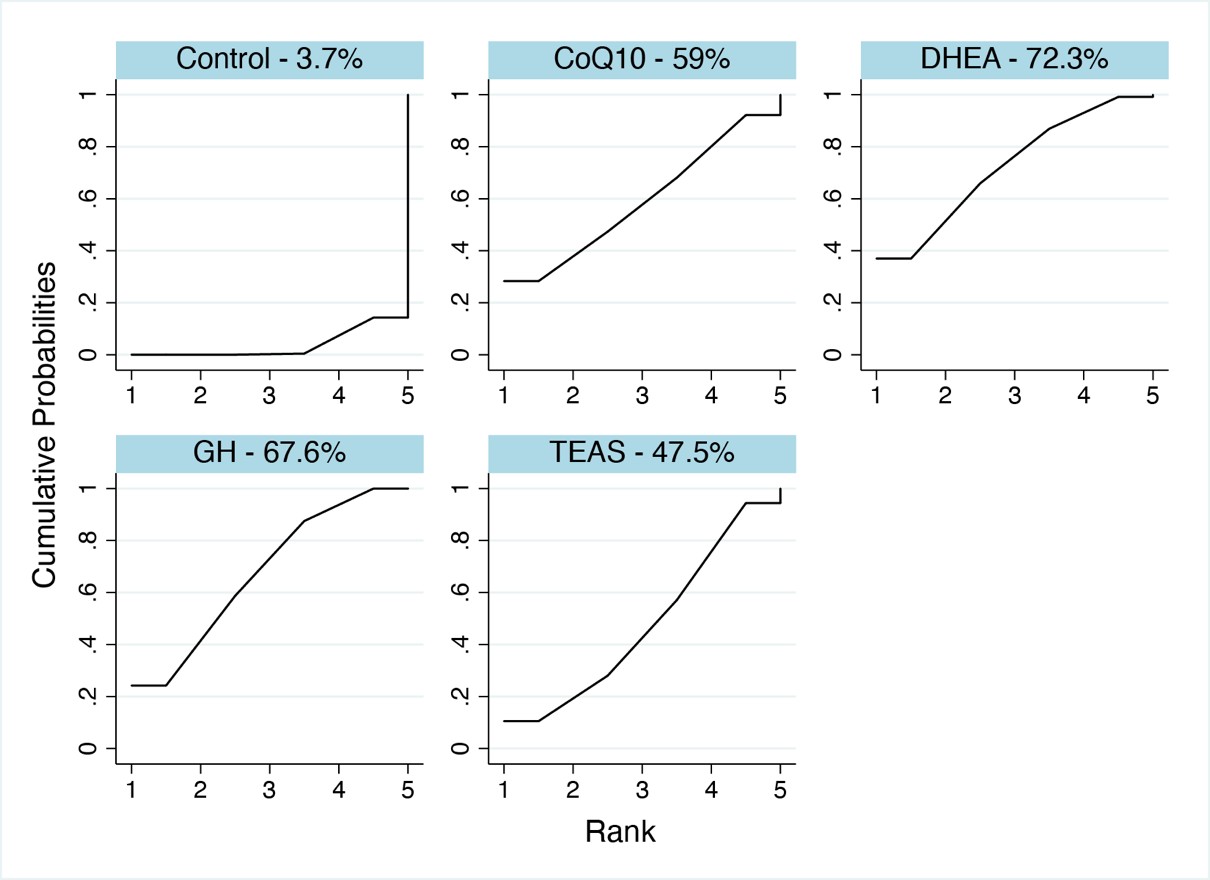
**
